# Next-Gen Medical Imaging: U-Net Evolution and the Rise of Transformers

**DOI:** 10.3390/s24144668

**Published:** 2024-07-18

**Authors:** Chen Zhang, Xiangyao Deng, Sai Ho Ling

**Affiliations:** School of Electrical and Data Engineering, University of Technology Sydney, Ultimo, NSW 2007, Australia; chen.zhang-6@student.uts.edu.au (C.Z.); xiangyao.deng@student.uts.edu.au (X.D.)

**Keywords:** medical imaging segmentation, deep learning, Transformer-based models, medical sensing, X-ray, CT scan, ultrasound device, high resolution, sensitivity, noisy level

## Abstract

The advancement of medical imaging has profoundly impacted our understanding of the human body and various diseases. It has led to the continuous refinement of related technologies over many years. Despite these advancements, several challenges persist in the development of medical imaging, including data shortages characterized by low contrast, high noise levels, and limited image resolution. The U-Net architecture has significantly evolved to address these challenges, becoming a staple in medical imaging due to its effective performance and numerous updated versions. However, the emergence of Transformer-based models marks a new era in deep learning for medical imaging. These models and their variants promise substantial progress, necessitating a comparative analysis to comprehend recent advancements. This review begins by exploring the fundamental U-Net architecture and its variants, then examines the limitations encountered during its evolution. It then introduces the Transformer-based self-attention mechanism and investigates how modern models incorporate positional information. The review emphasizes the revolutionary potential of Transformer-based techniques, discusses their limitations, and outlines potential avenues for future research.

## 1. Introduction

For medical segmentation, data scarcity has long been a persistent challenge. Unlike natural images, annotating medical image data often necessitates the expertise of trained professionals, making data collection a complex endeavor. Additionally, scaling up data collection for rare medical cases proves to be a difficult task. Ethical and privacy considerations further complicate the aggregation and sharing of medical data [1,2]. Compounding these challenges are the inherent features of medical images, including low contrast, high noise levels, and limited image resolution. These factors, particularly evident in ultrasound image segmentation, have posed significant barriers to achieving precise and reliable results, as reported in recent research by [3,4,5]. However, with its rapid development, traditional convolutional algorithms have encountered bottlenecks, necessitating further technological innovations to enhance efficiency [6].

Recent years have revealed the convergence of deep learning and computer vision, presenting transformative opportunities for medical imaging. With their self-attention mechanisms, the introduction of Transformer-based models has demonstrated the ability to produce promising results, especially in handling longer-range content [7]. This breakthrough offers the potential to recognize global information and represents a technological stride beyond existing bottlenecks [5,8].

In this literature review, we embark on a deep exploration of articles within this specialized domain. The review begins with a detailed examination of the historical evolution of medical imaging, shedding light on its inherent difficulties and challenges. Subsequently, we delve into a comprehensive assessment of specific technologies. The focus then shifts to an in-depth analysis of Transformer-based approaches, particularly the self-attention mechanism. Furthermore, we investigate cutting-edge advancements, such as incorporating positional information into algorithms.

Finally, this comprehensive review aims to provide significant insights into the most recent advancements and technological innovations, emphasizing the revolutionary potential of Transformer-based approaches in transforming the future landscape of medical imaging.

## 2. Medical Imaging Segmentation

Doctors in the traditional healthcare system rely primarily on their quick cognitive capabilities to guide complex treatments. In contrast, computer vision in the modern medical system evaluates medical data, such as images, using machine learning, deep learning, and other technologies, thereby supporting doctors in making high-accuracy medical decisions [2,9]. However, there has always been a need for more data in medical images. Annotation of medical image data requires experienced specialists, making it resource-intensive [10]. Additionally, it is challenging to scale data in unusual circumstances, and factors such as ethical privacy complicate aggregate data disclosure [4]. Furthermore, standard medical imaging technologies such as computed tomography (CT), magnetic resonance imaging (MRI), X-ray, and ultrasound have limitations, including low contrast and high noise.

### 2.1. Difficulty from Imaging Sensor

In medical imaging, an imaging sensor is a device used to capture light and convert it into electrical signals [11]. It is crucial in modern medical imaging equipment such as X-ray machines, CT scanners, MRI machines, and ultrasound devices [12,13,14]. Due to the imaging theory [15], in the medical field, it has several characteristics:

- High resolution: the number of pixels of the sensor is crucial, as a higher resolution allows for more detailed images, which is essential for accurate diagnosis;
- High sensitivity: the sensor’s performance in low-light conditions ensures that high sensitivity provides clear images even with low radiation doses, enhancing patient safety;
- High noise level: the random electrical signals generated during image capture need to be minimized since lower noise levels lead to clearer and more accurate images, reducing the likelihood of misdiagnosis.

### 2.2. U-Net and Its Variants’ Structures

**Figure 1:**
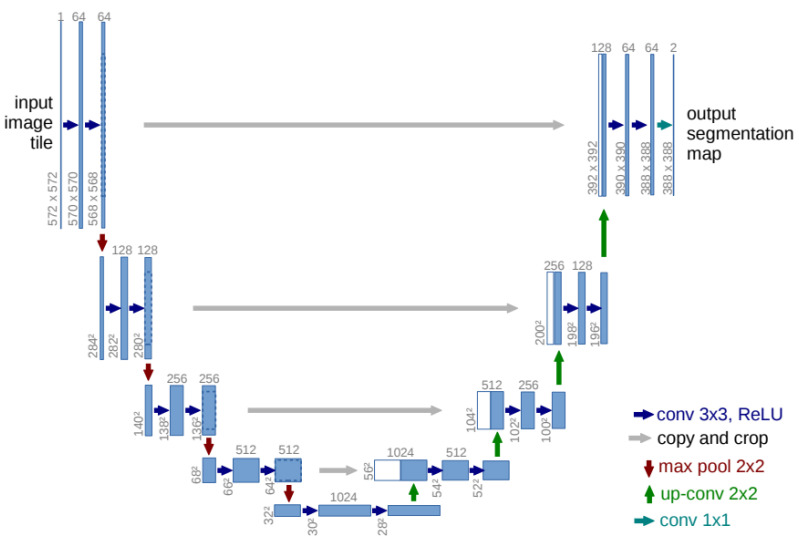
The U-Net architecture has a contracting path on the left and an expansive path on the right. The contracting path consists of repeated 3×3 convolutions with ReLU and 2×2 max pooling for downsampling, doubling the feature channels at each step. The expansive path involves up-convolutions and concatenations with cropped feature maps from the contracting path, ending with a 1×1 convolution to produce the output segmentation map [6].

The U-shaped Network(U-Net) architecture (as shown in Figure 1) and its variants are widely favored in medical image segmentation due to their exceptional performance, adaptability, and efficiency [10]. They excel in organ delineation, tumor detection, and cell counting tasks, offering state-of-the-art results in various medical imaging challenges [16]. U-Net’s fully convolutional design accommodates varying image sizes, and its incorporation of skip connections enables it to capture high- and low-level features crucial for precise results. Moreover, its ability to perform well with limited training data and its real-time inference capabilities make it practical for clinical applications. The openness and interpretability of U-Net further cement its popularity and impact in advancing medical image analysis and diagnosis [17,18]. Therefore, most articles in the field focus on this architecture.

However, as research questions become more complex, especially considering the extraordinary and unpredictable complexity of medical images, the basic U-Net architecture has encountered technical bottlenecks [19,20,21,22]. Specifically, traditional U-Net models face challenges in the backpropagation process, such as gradient disappearance, feature loss, and uneven response. There are several typical variants based on it.

#### 2.2.1. Residual Module

**Figure 2:**
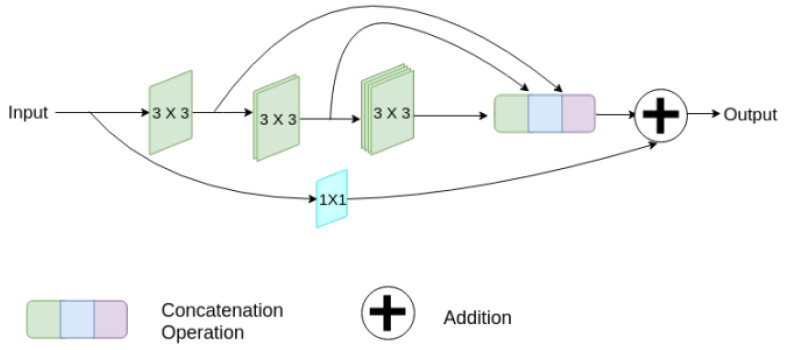
A diagram of the strategy, which involves gradually increasing the overall amount of filters in each of the three succeeding layers while also introducing a residual connection [23].

Innovated by residual learning [23], many models have incorporated residual elements into their architectures. Similar to the basic U-Net, these models also feature encoder and decoder pathways and skip connections. The initial motivation for this structural design was to address issues such as vanishing gradients in deep neural networks during training [23]. Vanishing gradients occur when the gradients of the loss function toward the network’s parameters become very small as they are back-propagated through many layers. This phenomenon can hinder convergence or even prevent effective learning, especially in intense neural networks. Residual networks mitigate this problem by introducing “skip connections” or “residual connections”, allowing information to bypass or be added to the middle layers (as Figure 2). ResPath is widely used in skip connections. Along these lines, many models have achieved improved results in specific applications, such as SIU-net for ultrasound spine image segmentation, MultiResUNet, Multi-Scale U-Net, and RSU-Net for cardiac magnetic resonance image segmentation [3,24,25,26], among others.

ResUNet (Residual and U-Net), introduced by [27], is a typical example of combining the residual network and U-Net. Building upon ResUNet, ResUNet++ [28] underwent further modifications. This model not only utilizes skip connections to pass feature maps of different scales but also incorporates channel attention weights. In this way, the model can use this weighted information to filter unnecessary details in the decoder feature map before passing it to subsequent network layers.

#### 2.2.2. Intention Module

As the difficulty of tasks increases, the ability to focus on specific targets or objects while ignoring irrelevant areas becomes crucial. Models can handle more challenging tasks effectively through selection gates [29,30]. The selection gate, commonly used in expansive and contracting paths and skip connections, is one of the most common applications of attention modules. It significantly improves segmentation results without adding unnecessary computational complexity.

For instance, the attention gate is widely used in various image tasks. For example, ASCU-Net utilizes an attention gate for skin lesion segmentation [31], while another variant enhances U-Net for abnormal tissue segmentation using a spatial attention gate [32]. Furthermore, Attention U-Net++ employs Attention U-Net for liver CT imaging segmentation [33], among other applications.

In practice, many networks utilize multiple modules tailored to their specific tasks. For example, RAD-UNet is introduced to minimize streak artifacts in CT images reconstructed from sparse-view projections, improving reconstruction accuracy and preserving image details [34]. LDS U-Net (Light-convolution Dense Selection) was designed for segmenting ultrasound bony features [35]. Additionally, DRAUNet, a deep network with a biplane joint method, enhances liver area segmentation from CT scans by incorporating 3D spatial information [36]. Moreover, models like DENSE-Inception U-Net [37] tackle more complicated segmentation tasks, while H-DenseUNET focuses on liver and tumor segmentation in CT scanning [38], and 2D Dense-Unet is applied for automated glioma segmentation [39]. In SIU-Net [3], a similar dense structure is also utilized for skip connection paths.

However, researchers have observed that increasing the number of network layers only sometimes leads to better segmentation performance in practice. Instead, it can lead to problems such as overfitting, a “black box” structure in the middle layers, and poor performance on long series of data [7,10,19,23]. Some networks may exhibit poor results on global content [40]. Consequently, this indicates a technical bottleneck in traditional U-Net models and their variants, requiring further technological innovation to address these issues and improve segmentation model performance.

## 3. Transformer in Medical Imaging

When introduced in 2017, the Transformer architecture, particularly its innovative self-attention mechanism, represented a significant milestone in deep learning [41,42,43]. This groundbreaking architecture brought about a considerable shift in how neural networks handle sequential and structured input, initially developed for applications in natural language processing [41,44,45]. The Transformer introduced a parallelized and attention-driven approach to processing sequences, contrasting earlier recurrent neural networks (RNN) and convolutional neural networks (CNNs) [46,47]. It not only dramatically improved model training efficiency but also achieved state-of-the-art results across various natural language understanding and generation tasks [48,49,50,51].

**Figure 3:**
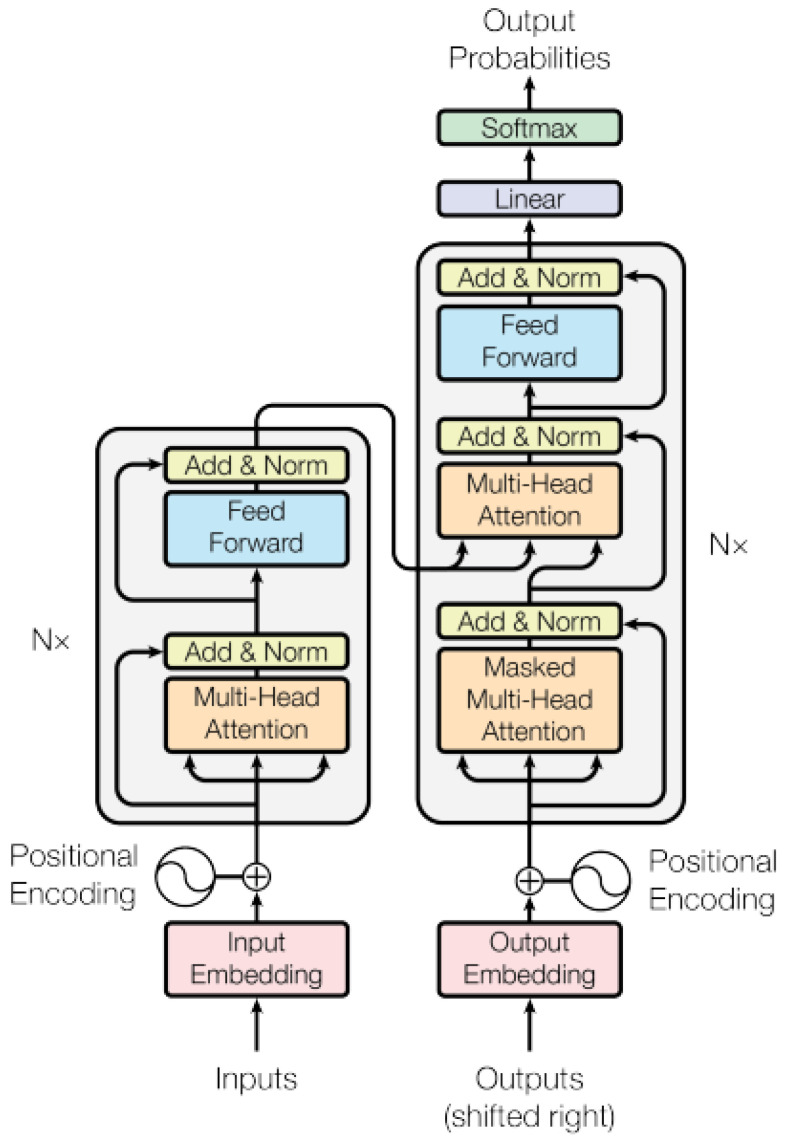
The Transformer structure, consisting of an encoder and a decoder as its primary components, each comprising multiple layers [41].

The Transformer architecture consists mainly of an encoder and a decoder [41]. Each encoder comprises components such as position coding, a multi-head self-attention mechanism, layer bormalization (LN) [52,53], a feed-forward network (FFN) [54], and fully connected layers. The decoder structure is similar to that of the encoder but includes a masked multi-head self-attention mechanism at the input layer [55,56]. The architecture is illustrated in Figure 3.

Two key components stand out in grasping the essence of the Transformer architecture. Among them, the self-attention mechanism plays a paramount role [41]. There are three main components: Queries (Q), Keys (K), and Values (V) [57,58,59]. This mechanism aims to calculate a weighted sum, allowing information aggregation from the Values into the Queries.

Whenever an algorithm calculates an attention score, several key steps are involved in the process [41,60]:

1. The algorithm will initialize the matrix for K, Q, and V.
2. The relationship between Queries and Keys: Each Query must first be connected to every Key. First, to facilitate the dot product operation, the algorithm transposes the K matrix and then multiplies the transposed matrix by the Q matrix. This indicates that each Query computes its correlation with each Key to determine which Keys are more pertinent to a given Query.
3. Scaling: The results of the correlation calculations are ordinarily divided by the square root of dk ($\sqrt{dk}$) to ensure consistent computations. This step helps to control the range of values to ensure the stability of the calculation.
4. Softmax: The Softmax function is applied to convert the correlation distribution of Query to Keys into a weight distribution. The Softmax function ensures that the sum of these weights is equal to 1, and the appropriate weights are assigned according to the strength of the correlation.
5. Multiplication of Weights with Values: These weights are multiplied by the corresponding values. This step weights the Query information with the related Key details to produce the final output.

(1) $$\mathrm{Attention}\left(Q,K,V\right)=\mathrm{Softmax}\left(\frac{QK^{T}}{\sqrt{d_{k}}}\right)V$$

**Figure 4:**
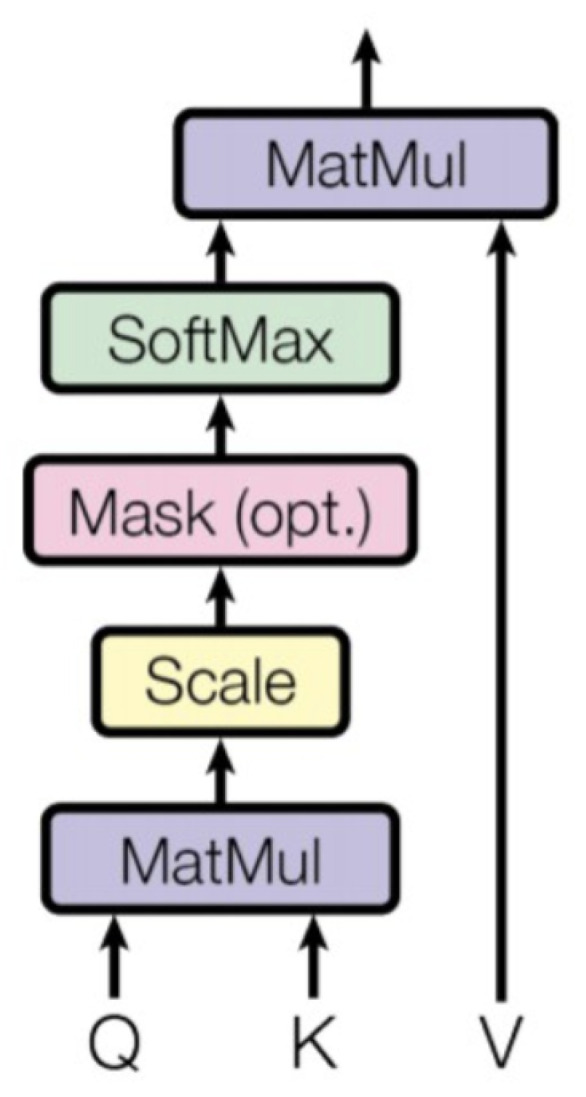
The key mechanism in the Transformer architecture, which computes attention scores between elements in a sequence, providing a weighted representation of the input [41].

Consequently, the score in this attention mechanism signifies a measure of correlation or similarity between a Query and a set of keys [41,60]. These scores indicate how strongly the model focuses on different locations or elements based on the correlation between the Query and the Key (Equation (1)), facilitating effective weighted input data aggregation. This is one of the reasons why the self-attention mechanism finds utility across various tasks, as it captures complex relationships and dependencies in data (Figure 4).

### 3.1. Transformers in Computer Vision

Although the first significant application of the Transformer module is in natural language processing, many studies have found that the Transformer module can also be used in the computer vision area [40]. Incorporating the Transformer’s self-attention mechanism has significantly changed how visual data are processed and understood in computer vision. CNNs were primarily used in traditional computer vision methods for picture classification, object recognition, and image segmentation. But with its attention-based mechanism, the Transformer architecture has ushered in a new age for managing visual data [61,62].

One of the pioneers in this area is the vision Transformer (ViT) [63]. It is a deep learning model introduced by Alexey Dosovitskiy and his team in 2020, marking a significant advancement in computer vision. ViT took a fresh approach by dividing an image into smaller patches, arranging them into a sequence, and using the Transformer’s self-attention mechanism to capture the relationships and dependencies between them. This innovative shift delivered impressive performance in image classification. It opened the doors for the widespread adoption of Transformer-based models in computer vision, establishing ViT as a significant milestone in the field. It plays an essential role in the areas of self-attention and computer vision. Based on that [63], the Swin Transformer was invented for multi-scale reception fields [8]. Furthermore, there is another attempt to use ViT in breast cancer screening [64] and the deeper Vision Transformer in similar segmentation applications [65].

#### Self-Attention and Convolutional Operation

Compared to regular convolutional operations, the self-attention mechanism emphasizes global context information more strongly. It achieves comprehensive connectivity by modeling relationships between all elements [61]. This means that each component can affect all the others, better capturing more global information. Moreover, it excels at capturing intricate relationships and dependencies across distant positions in sequential data, making it a potent tool for handling long-range dependencies.

In Table 1, the advantages and drawbacks of the self-attention mechanism and convolutional operation [61] are highlighted. The self-attention mechanism is better suited for processing sequence data, especially with long-distance dependencies. Meanwhile, the convolution operation is better for local features because it can effectively capture local structures and features in an image. Therefore, there is a new trend of combining the advantages of two different structures so the model can perform well in long-range sequence information extraction and local information extraction. Regarding the medical imaging process, this provides more chances for applications like segmentation, especially noise removal.

### 3.2. Feature Extraction

There are several ways to combine transformers. RNN and long short-term memory (LSTM) are more position sensitive than bon-recurrent models, which incorporate position bias by loading the input tokens in a sequential order [66]. The primary cause of this issue is that position information among input units is not intrinsically encoded; as a result, they are identical in permutations. This issue explains why every known model includes a position encoding/embedding layer at the input. Similarly, Transformer-based models also require careful consideration of positional information. As one of the examples, ViT already includes position embedding with the patch for adding positional information [41], but the position information is still relatively weak.

#### 3.2.1. Hybrid Structure

The first way to implement a combination is through a hybrid structure.Most applications in this area simply combine the advantages of both convolutional operation and self-attention with a hybrid structure. As a result, this is the most common combination technology [67].

TransBTS is a unique neural network architecture for MRI brain tumor segmentation, seamlessly combining Transformer and the 3D CNN, effectively capturing local and global characteristics. CoTr integrates multi-scale feature maps and employs a 3D deformable Transformer with a bridge module to enhance feature fusion and attention computation while reducing computational complexity [68,69]. AFTer-UNet addresses axis information in 3D volumes and achieves superior segmentation accuracy with fewer parameters compared to previous Transformer-based models [70].

TransUNet, another innovative hybrid structure, combines Transformer and the 3D CNN, effectively capturing local and global characteristics. It outperforms previous state-of-the-art 3D algorithms for brain tumor segmentation in 3D MRI scans [7]. HiFormer efficiently combines a CNN and a Transformer, leveraging multi-scale feature representations and introducing a double-level fusion module to fuse global and local features [71].

To address uncertainties, TransUNet+ enhances skip features using a Transformer block’s score matrix to improve global attention, achieving superior performance, especially in tiny organ segmentation [72]. H-TUNet integrates a multi-scale cross-attention Transformer module, effectively capturing anatomical distinctions and enhancing contextual features, demonstrating exceptional performance in thyroid disease diagnosis [73].

DA-TransUNet is a novel deep medical image segmentation framework that integrates Transformers and dual attention blocks (DA-Block) into a U-shaped architecture [74], optimizing position and channel features to enhance feature extraction and performance, consistently outperforming state-of-the-art techniques across five datasets.

ScribFormer, a new Transformer-CNN hybrid solution, achieved superior segmentation performance over state-of-the-art scribble-supervised methods on the ACDC, MSCMRseg, and HeartUII datasets [75]. It demonstrated new state-of-the-art (SOTA) performance on the ACDC, MSCMRseg, and HeartUII datasets.

There are numerous models with similar structures, and one of the most typical and commonly studied approaches involves combining the basic CNN or its variants with Transformers and self-attention mechanisms. This category is characterized by its straightforward integration of these components.

#### 3.2.2. Self-Attention Block

Another implementation approach involves incorporating the self-attention mechanism into the feature selection block. It replaces the standard convolutional operation with a Transformer within the U-Net architecture. It is evident that there are also several hybrid variants; however, unlike that mention in the previous chapter, the convolutional operation is entirely replaced by the self-attention mechanism.

Based on the Swin Transformer [8], one of the essential models in the medical field is called Swin-Unet [76]. Since the traditional CNN network has long-range semantic information extraction limitations, Swin-Unet is intended to be a pure Transformer similar to Unet for medical picture segmentation. The tokenized image patches are fed into a U-shaped encoder–decoder architecture based on Transformers and skip connections. Furthermore, to compensate for local information, a window-based system was introduced.

**Figure 5:**
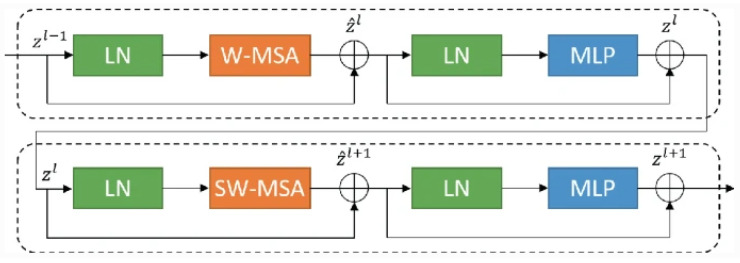
The Swin block contains two consecutive blocks incorporating windowed multi-head self-attention (W-MSA) and offset windowed multi-head self-attention (SW-MSA) modules [41].

As Figure 5 illustrates, the LN layer, multi-head self-attention module, residual connection, and two-layer MLP with GELU nonlinearity are all included in each Swin Transformer block. The windowed multi-head self-attention module (W-MSA) and the offset windowed multi-head self-attention module (SW-MSA) are employed in two consecutive Transformer blocks. The precise examples for W-MSA and SW-MSA are as follows:

- W-MSA: In W-MSA, input data are divided into windows, each containing multiple adjacent blocks of data. Self-attention operations are performed in each window, allowing each block of data to perform self-attention calculations with other blocks in the same window. This helps capture local features.
- SW-MSA: SW-MSA is an improved multi-head self-attention mechanism that introduces the offset of the window. This means that when calculating self-attention, it is no longer limited to the data blocks within the window but takes into account the relationships between the windows. This helps capture a wider range of contextual information.

The Swin Transformer, known for its window-based self-attention mechanism and shifted window mechanism, divides input data into windows to capture local features effectively [76]. This model has significantly advanced deep learning, with its ability to process local and global information. Improved versions have shown remarkable performance on public medical datasets.

For instance, ST-Unet combines the Swin Transformer as an encoder and CNNs as a decoder, introducing a cross-layer feature enhancement (CLFE) module and a spatial and channel squeeze and excitation module to improve feature learning across different layers and highlight specific areas’ importance [77]. TransDeepLab, a pure Transformer for medical image segmentation, employs Swin Transformer blocks to capture local and long-distance context information, integrating multi-scale features into the decoder through cross-contextual attention mechanisms [78]. TransConver replaces the multi-branching structure in GoogLeNet with Transformer modules and convolutional modules, facilitating interactions between global and local features, thus improving tumor segmentation accuracy and reducing computational load [79].

CSwin model combines CNN and Swin blocks to leverage both models’ advantages, integrating the interactive channel attention (ICA) module, gating-based auxiliary feature fusion (SFF) module, and boundary detection (BD) module to improve breast lesion segmentation performance [80]. Another approach involves entirely modified versions, such as PCAT-UNet, incorporating cross-patch convolution self-attention (CPCA) and inner patch convolution self-attention (IPCA) modules, and MT-Unet, introducing the hybrid Transformer module (MTM) for intra- and inter-sample affinity relationships [81]. These methods have demonstrated superior performance on diverse medical image datasets. Other than that, SSTrans-Unet highlights the limitation of fixed masks in the Swin Transformer and represents a novel approach that can better capture long-range dependencies channel-wisely [82].

To summarize this model’s applications, it has the following characteristics:

- Transformer position selection, which affects model performance: Choosing a segmentation model that places the Transformer in the encoder is more common than a segmentation model that places it in the decoder. This is because encoders are mainly used to extract features, while decoders are used primarily to fuse features extracted by encoders.
- Feature expression ability improvement: In order to better fuse global and local information, it is common to use a Transformer in the encoder to extract information and then use a Transformer in the decoder to fuse the information and combine the convolutional network to obtain detailed features as an advantage, so as to enhance the model’s ability to express features.
- Complexity and efficiency trade-offs: Inserting Transformer modules into both the encoder and decoder increases the computational complexity of the attention mechanism, resulting in a decrease in model efficiency. Therefore, efficient attention modules need to be explored to improve the efficiency of such models.
- Balance at transition junctions: Placing the Transformer at the transition junction is a trade-off option to draw connections from features with low expressiveness while relying on global features to guide subsequent fusions. This is because the feature map at the transition junction has the lowest resolution, and even if you use a multi-layer superimposed Transformer module, it will not put a large load on the model. However, this approach has limited capabilities in feature extraction and fusion, and there is a trade-off between its use and its costs.

Therefore, placing transformers in different parts has different benefits; the selection should be based on their applications, balancing the benefits and drawbacks.

#### 3.2.3. Others

Several innovative approaches have emerged to tackle specific challenges in medical image segmentation by integrating attention mechanisms and transformer architectures into encoder–decoder frameworks. Models like EG-TransUNet, TransCeption, HiFormer, HTNet, and RTNet [71,83,84,85,86] leverage multi-head self-attention, Transformer-enhanced modules, multi-scale feature extraction and fusion, dual-Transformer bridging, position-sensitive axis attention, and relational Transformer modules to improve feature discrimination, capture global context, fuse spatial and semantic information effectively, and model relationships between regions and lesions [66]. These approaches have shown impressive performance in various medical image segmentation tasks, surpassing traditional CNN-based and hybrid methods in both quantitative and qualitative results. MultiTrans introduces a novel multi-branch Transformer (MultiTrans) architecture with a memory- and computation-efficient self-attention module to address the challenges of using Transformer models for medical image segmentation [87].

**Figure 6:**
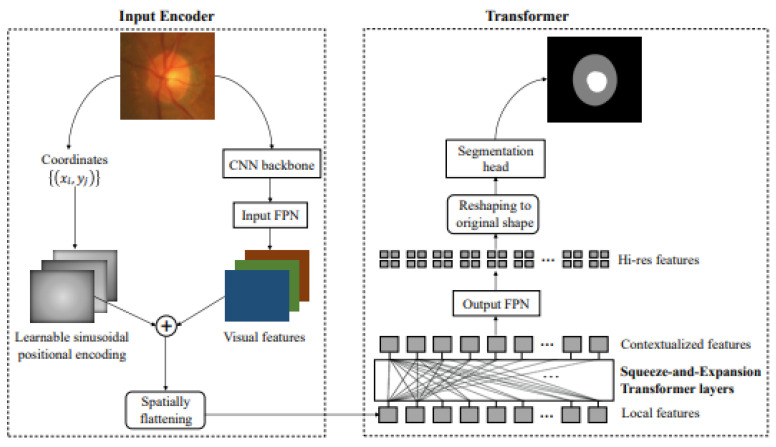
A novel positional encoding scheme customized for images with a continuity-inducing bias is integrated with pitch coordinates and CNN-based extraction [88].

Furthermore, there is a novel segmentation framework based on Transformers called Segtra [88]. Transformers have the advantage of having an infinite number of effective receptive fields, even at high feature resolutions. The development of a unique squeeze-and-expansion Transformer, which contains both squeezed attention blocks to regularize self-attention and expansion blocks to acquire varied representations, is the key innovation within Segtran (Figure 6).

Some follow-up works have explored improvements in Transformer-based models by incorporating fixed or learned positional encoding methods. For example, location information can be computed as an embedding matrix using sine or cosine functions and added to the attention algorithm [89]. Another approach involves absolute position encoding, integrating segment encoding and relative positional information into the token attention matrix [90,91]. Furthermore, the latest work, SegFormer3D, is a lightweight hierarchical Transformer for 3D medical image segmentation that efficiently calculates attention across multi-scale features with an all-MLP decoder, achieving competitive performance on key datasets with significantly fewer parameters and lower computational requirements compared to state-of-the-art models [92].

Overall, Transformers have been seamlessly integrated into various network architectures, enhancing their capabilities and significantly improving performance across diverse domains by capturing boundaries, enhancing targets, refining feature processing, and more.

#### 3.2.4. Summary

In Transformer-based networks within the medical field, the evolution of positional coding methods has traversed distinct phases in recent years. With the advent of the ViT in 2021, the focus of research in 2021 and 2022 revolved around the development of intricate mathematical transformations and hybrid architectures that seamlessly fused convolutional processes with transformer units, as evidenced in notable works such as [70,88,93,94]. These efforts were motivated by a need to address the basic limits of the self-attention mechanism, specifically its processing of local information. However, the landscape underwent a substantial transformation with the introduction of the Swin Transformer in 2022. This significant advancement signaled a shift in emphasis toward the use of fixed or shifting window-based positional coding techniques. It is important to note that position-encoding structures prioritized mathematical implementation, whereas hybrid structures combined convolutional processes with the transformer mechanism’s transformative power. The self-attention block evolved as a revolutionary strategy that primarily used self-attention for feature extraction, frequently combining hybrid structures for improved performance and focusing on Transformer-based blocks and other convolutional processes [95].

### 3.3. Learning Strategy

Although the transformer-based model has been implemented for several years and generated good results, there are still several further performance enhancement strategies:

#### 3.3.1. Semi-Supervision

Combining semi-supervised learning with Transformers: Semi-supervised learning is a widely used method in machine learning to address the challenge of limited labeled samples in datasets [96,97,98], particularly in the field of medical imaging where obtaining large-scale labeled samples is often tricky [4,99,100]. Leveraging the exceptional capabilities of Transformer models, semi-supervised learning can effectively utilize large-scale unlabeled medical image data by automatically generating high-confidence pseudo-labels to expand the training dataset. This approach enhances the model’s generalization ability and better handles the diversity and complexity present in medical images [101,102]. Combining semi-supervised learning with Transformers promises to provide more accurate solutions for medical image analysis, with potential improvements in medical applications such as disease diagnosis, medical image processing, and patient care. This method fully capitalizes on medical image data while addressing the challenges of data scarcity in the medical field. While there are existing approaches, further research is needed to fine-tune the handling of specific medical image processing tasks. Additionally, due to the unique characteristics of the self-attention algorithm [63,103], the more training iterations it undergoes, the more accurate the results it can generate. Therefore, having more training data, including semi-supervised data, can be beneficial for improving performance [76]. One possible solution is contrastive learning [104,105,106]. Combining extra learning tasks may increase the model performance by distinguishing each class and benefiting the general model segmentation performance.

#### 3.3.2. Class Awareness Enhancement

Another alternative to enhancing the model is by improving class differentiation, such as with the class-aware adversarial Transformer [102]. This approach constructs multi-scale representations, handles multi-scale variations, and uses a novel class-aware Transformer module to learn discriminative regions of objects with semantic structures. Similarly, ClassFormer employs similar concepts to address intra-class and inter-class issues within specific medical image tasks [107]. Another example is the hierarchical class-aware domain adaptive network [108], which integrates an anisotropic neural network and a Transformer (AsTr) to extract multi-scale context features from CT images with an anisotropic resolution. It includes a hierarchical class-aware domain alignment (HCADA) module to adaptively align these features across domains using a class attention map. In conclusion, class-aware enhancement with Transformers improves segmentation accuracy by incorporating class-specific information into the Transformer model. A class-aware Transformer module is used to enhance the learning and differentiation of the discriminative regions of objects based on their semantic structures.

#### 3.3.3. Uncertainty Awareness

Another challenge in medical image segmentation is annotation accuracy; due to labeling issues, accurately annotating data is time-consuming and difficult. Therefore, uncertainty prediction has become prominent. UCTNet’s uncertainty-guided Transformer module (UgViT) effectively minimizes the functional overlap between CNN and Transformer, leading to superior performance compared to other hybrid approaches [109]. Its computational complexity is reduced compared to vanilla Transformer-based approaches due to the selective application of self-attention on uncertain regions. Another example is the Semi-supervised network model for contrastive learning based on entropy constraints [110]. This model introduces a semi-supervised learning method for CT image segmentation that combines CNN and Transformer models with entropy-constrained contrastive learning, improving performance with less labeled data through uncertainty awareness. Other notable examples include Rectified Contrastive Pseudo Supervision and Uncertainty-aware Representation Calibration [111,112].

## 4. Discussion and Limitation

The articles discuss recent advancements in medical imaging, with a primary emphasis on medical image segmentation. Given the prominence of the U-Net architecture in this domain [10,113,114,115], the articles commence by exploring several specialized architectural components. These components include residual, inception, dense, and attention modules, which play a significant role within the U-Net framework and find applicability in other deep learning networks, notably the Transformer architecture. Furthermore, the central portion of the articles is dedicated to discussing Transformer-based U-Net architectures. Additionally, the articles highlight self-attention mechanisms, convolutional processes, positional encoding techniques, and the integration of hybrid structures.

While the Transformer-based design has significantly contributed to computer vision, particularly in medical image processing, its limits remain clear. Below are a few examples of where these restrictions become apparent:

- Data limitations: Medical image datasets are more difficult to obtain than ordinary computer vision datasets [4]. The issues involved are more complex, including privacy concerns, data scarcity, diversity (such as X-rays, MRIs, CTs, ultrasounds, etc.), and the specialization of the medical field (which usually requires annotation by professional doctors). This data limitation poses a significant challenge for Transformer-based models, as they heavily rely on the self-attention mechanism to capture long-range dependencies and global context information [114]. The self-attention algorithm’s complexity scales quadratically with the input sequence length, making it computationally expensive, especially for high-resolution medical images. Consequently, Transformer-based models require larger datasets to learn the intricate patterns and relationships within medical images [116]. However, the scarcity of annotated medical data can hinder the model’s ability to leverage the self-attention mechanism fully, potentially limiting its performance compared to that of CNN, which is more parameter-efficient and can better generalize from smaller datasets.
- Generalization: Generalization is a prevalent concept in developing deep learning, particularly within computer vision, where large pre-trained models are commonplace [117]. These models are characterized by their extensive parameter count and intricate architecture. Among these, Transformer-based large models stand out as a prime example. They can adapt to various datasets within their respective domains with minimal effort, necessitating only fine-tuning for different applications [118,119]. This flexibility enables seamless migration from one task to another, eliminating the need for excessive additional training. However, the medical field faces a unique challenge in adopting pre-trained large models. This is primarily attributed to the intricacies and lack of medical data, making developing such models a formidable endeavor.

## 5. Conclusions and Research Direction

Medical image segmentation, a crucial application of computer vision in healthcare, initially saw great success with CNN models, particularly U-Net. U-Net’s encoder–decoder structure and skip connections effectively capture multi-scale image features. Researchers have since enhanced U-Net with modules like residuals to address vanishing gradients, inception for multi-scale features, and dense connections for more layer interaction, improving segmentation in complex scenarios. Recently, Transformers have been introduced, combining self-attention mechanisms with U-Net to model long-range dependencies and improve global feature learning. While promising, Transformer-based models in this field are still emerging and face challenges like data scarcity and generalization. Future innovations are expected to tackle these complexities further.

The following research directions are based on current research on Transformers, self-attention processes, and U-Net fusion:

- Multi-scale feature extraction: In deep U-shaped networks, the upper model first learns broader features such as edges and textures, and as the network increases, the underlying structure extracts higher-level features. Then, at this time, the data are transferred between different levels, and data loss will inevitably occur [120]. This is also the reason why the model is less effective for segmentation. However, the previous model also has techniques such as regional feature enhancement or hierarchical feature jumping [72,80,83], although further research must emphasize enhancing edge detection and noise cancellation. One of the position directions could be federal learning, combining different reception fields to generate a more comprehensive result [121].
- Further local–global context extraction: To further enhance local and global information extraction, integrating advanced methods such as hybrid models that combine CNNs with Transformers can be promising. These models can leverage the strengths of CNNs in capturing fine-grained local features and the capability of Transformers in modeling long-range dependencies [122]. Additionally, incorporating multi-head self-attention mechanisms and hierarchical attention structures can improve the model’s ability to capture nuanced details and broader contextual information simultaneously [41]. Techniques such as attention gating can also selectively focus on relevant parts of the image, enhancing the overall segmentation accuracy [29]. Moreover, combining these methods with advanced data augmentation techniques and synthetic data generation can address the data scarcity issue and further improve the robustness and generalization of the models in medical image segmentation.

Image processing model creation is basic in computer vision and artificial intelligence. These models are crucial in tasks ranging from object recognition to medical image segmentation. Transformers play an essential role in the medical field. They are superior at capturing global context and instrumental in complex tasks such as medical image segmentation. On the other hand, small medical picture datasets present difficulties for Transformers. U-shaped networks that are efficient for image tasks are introduced to address this. They achieve an integration of global and local information. This paper investigates using Transformers and U-shaped networks in medical picture segmentation to increase performance. The outcomes demonstrate their synergy in tackling these problems.

## Figures and Tables

**Table 1:** Summary of advantages and disadvantages of self-attention mechanism and convolutional operation.

Characteristics	Self-Attention Mechanism	Convolutional Operation
Applicability	Suitable for long-range dependencies.	Suitable for extracting local features and structures.
	Fully connected; each element can influence all others.	Locally connected; each neuron relates to a small portion of the input.
Parameter Count	More parameters; requires more computational resources.	Fewer parameters; more computationally efficient.
Computational Efficiency	Higher computational complexity.	Lower computational complexity, particularly for large-scale data.
Translation Invariance	Lacks translation invariance; sensitive to position.	Possesses translation invariance; insensitive to position.
	Often requires position encoding for handling sequence information.	No need for additional position encoding.

## Data Availability

Data are contained within the article.
